# Genome Sequence of a Toxin-Positive *Clostridium difficile* Strain Isolated from Murine Feces

**DOI:** 10.1128/genomeA.00088-17

**Published:** 2017-04-06

**Authors:** Lucie Etienne-Mesmin, Benoit Chassaing, Oluwaseyi Adekunle, Lisa M. Mattei, Adrianne N. Edwards, Shonna M. McBride, Frederic D. Bushman, Andrew T. Gewirtz

**Affiliations:** a Center for Inflammation Immunity and Infection, Institute for Biomedical Sciences, Georgia State University, Atlanta, Georgia, USA; b Immunology and Molecular Pathogenesis Graduate Program, Emory University, Atlanta, Georgia, USA; c PennCHOP Microbiome, Children’s Hospital of Philadelphia, Philadelphia, Pennsylvania, USA; d Department of Microbiology and Immunology, Emory University School of Medicine, Emory Antibiotic Resistance Center, Atlanta, Georgia, USA; e Department of Microbiology, University of Pennsylvania School of Medicine, Philadelphia, Pennsylvania, USA

## Abstract

Herein, we report the genome sequence of a *Clostridium difficile* strain isolated from the feces of antibiotic-treated C57BL/6 mice. We have named this strain, which differs considerably from those of the previously sequenced *C. difficile* strains, LEM1.

## GENOME ANNOUNCEMENT

*Clostridium difficile* is a toxin-producing spore-forming bacterium representing the leading cause of nosocomial diseases in the United States (1, 2). *C. difficile*-induced disease is largely driven by the two exotoxins A (TcdA) and B (TcdB), which are part of a 19.6-kb pathogenicity locus (PaLoc), that contribute to diarrhea and severe colitis (3). Ablation of the gut microbiota with the use of antibiotics renders individuals susceptible to *C. difficile* infection (CDI) (4). In mice, CDI is modeled by antibiotherapy (antibiotic cocktail in drinking water *ad libitum* and intraperitoneal injection of clindamycin), followed by exposure to *C. difficile* spores (5). We have observed that following this treatment, a substantial percentage of antibiotic-treated C57BL/6 mice exhibited detectable levels of fecal *C. difficile* prior to infection (6).

Fecal samples tested positive for *C. difficile* upon antibiotherapy were plated onto taurocholate-cefoxitin-cycloserine-fructose agar (7, 8) and incubated anaerobically (48 h, 37°C) (9). Isolates that exhibited the characteristic *C. difficile* colony morphologies were further isolated on brain heart infusion agar supplemented with yeast extract and subsequently tested PCR positive for the presence of the *tcdB* gene (data not shown). *C. difficile* spores were prepared as described by Edwards et al. (10).

Genomic DNA was extracted from a pure spore preparation of *C. difficile* LEM1, according to the Pacific Biosciences phenol-chloroform protocol (PacBio), with the following modifications prior to DNA extraction: 10 min of incubation with 10% SDS (64°C), 3 min of bead beating with glass beads, and centrifugation (12,000 rpm, 5 min). DNA integrity was checked by agarose gel electrophoresis, and purity was verified by 260 and 280 nm optical density measurements on an Epoch BioTek instrument.

Complete genome sequencing of *C. difficile* LEM1 was carried out using single-molecule real-time (SMRT) sequencing technology on a PacBio RSII (Pacific Biosciences, Menlo Park, CA). Each library was loaded on SMRT cells (SMRT Cells 8Pac version 3) and sequenced using C4 chemistry. Genome assembly was performed with the HGAP.2 protocol (SMRT Analysis 2.3.0). The resulting preassembled consensus sequences were quality trimmed and then passed to the Celera Assembler. The assembly was refined by aligning all reads back to the assembly using BLASR. Quiver was used to call highly accurate consensus sequences. The polished genome was annotated using the RAST pipeline (11). A single circularized chromosomal contig was generated, with 4,191,506 bp in length and a G+C content of 28.8%. *C. difficile* LEM1 contains 4,118 predicted coding DNA sequences and 123 RNA genes, among which 33 are rRNAs and 90 are tRNAs.

Comparisons with the well-characterized *C. difficile* strain VPI 10463 revealed high sequence similarity (91 to 95%) but also unveiled unique sequences in the genome of *C. difficile* LEM1. Genome analysis of LEM1 PaLoc, based on established standards within the *C. difficile* field (12, 13), revealed that this strain is toxinotype 0, like VPI 10463. There are no differences in the TcdA and TcdB sequences themselves, nor in their respective promoters. *In vivo* and *in vitro* assays demonstrated that LEM1 produced a smaller amount of toxins than VPI 10463 (6). Multiple mobile elements related to phages were detected in the *C. difficile* LEM1 genome and may play a role in the pathogenesis of this strain.

### Accession number(s).

This whole-genome shotgun project has been deposited at DDBJ/ENA/GenBank under the accession no. CP019469 for *C. difficile* LEM1 and MUJV00000000 for *C. difficile* VPI 10463. The versions described in this paper are versions CP019469.1 and MUJV01000000 (which consists of sequences MUJV01000001 to MUJV01000002) for LEM1 and VPI 10463, respectively.
